# The contribution of subthreshold preference in inhibitory neurons to network response

**DOI:** 10.1186/1471-2202-16-S1-P36

**Published:** 2015-12-18

**Authors:** Tatjana Tchumatchenko, Claudia Clopath

**Affiliations:** 1 Theory of Neural Dynamics Group, Max Planck Institute for Brain Research, Max-von-Laue-Strasse 4, 60438 Frankfurt, Germany; 2 Department of Bioengineering, Imperial College London, South Kensington Campus, London SW7 2AZ, UK

## 

**Figure 1:**
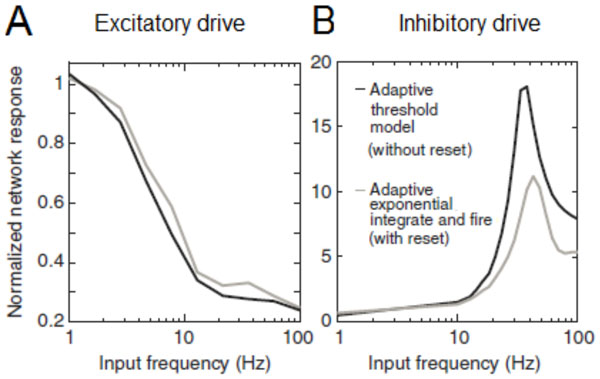
**Neuronal resonance in networks**. These results highlight that neuronal models which exhibit subthreshold preference, regardless of spike generator details, yield results that are consistent with experimental observations in [2]. (A) The response of a recurrent neural network, R_E_(2πf)/R_E_(0) to the stimulation of excitatory neurons with periodic stimuli of frequency *f *(B)The response of a recurrent neural network, R_I_(2πf)/R_I_(0), to the stimulation of excitatory neurons with periodic stimuli of frequency *f*. Figures adapted from [1].

Oscillations are one of the hallmarks of neural network activity. They have been implicated in numerous cognitive phenomena and have been observed in many brain regions. What is interesting about the mechanism of oscillations in the cortex is that inhibitory neurons seem to play an important role in the generation and maintenance of these global oscillations. Yet, is currently unclear what specific properties of these inhibitory neurons shape the rhythms. One hypothesis is that their intricate synaptic connections are essential. Alternatively, the intrinsic properties of the neurons themselves could be the main factor. In the study presented here and recently published in [1], we explore the combination of both aspects and consider a network model where interneurons are equipped with subthreshold resonance and electrical gap junctions. In contrast to previous studies we address the interaction of both gap junctions and subthreshold resonance in the same model network. The goal of our work is to offer a tractable mathematical description of how these two effects destabilize the fluctuation-driven state of a cortical network. In a cortical network neurons typically fire irregularly but nevertheless can, under some conditions, lead to a global resonance or synchronous oscillation. Here, we show that the oscillation frequency of this rhythm is determined by the single neuron resonance and modulated by the electrical and chemical synapses. We argue that the presence of both aspects, gap junctions and subthreshold resonance, facilitates for the emergence of oscillations in a cortical network. We find that our results are consistent with several experimental observations including network responses to oscillatory inputs in the barrel cortex, see Fig. 1. Thus, they provide a much-needed computational link connecting different, seemingly disparate effects that were previously observed in networks.
